# The Prospects for Payment for Ecosystem Services (PES) in Vietnam: A Look at Three Payment Schemes

**DOI:** 10.1007/s10745-012-9480-9

**Published:** 2012-04-01

**Authors:** Phuc Xuan To, Wolfram H. Dressler, Sango Mahanty, Thu Thuy Pham, Claudia Zingerli

**Affiliations:** 1 Forest and Nature Conservation Policy Group, Wageningen University, Wageningen, The Netherlands; 2 College of Asia and the Pacific, Australian National University, ACT 0200 Canberra, Australia; 3 Center for International Forestry Research, Bogor, Indonesia; 4 Climate-KIC Co-location Centre Switzerland ETH Zurich, Hochstrasse 60, HCH B14.1, 8092 Zurich, Switzerland

**Keywords:** Market-based mechanisms, Forest resources, Property rights, Poverty alleviation, Vietnam

## Abstract

Global conservation discourses and practices increasingly rely on market-based solutions to fulfill the dual objective of forest conservation and economic development. Although varied, these interventions are premised on the assumption that natural resources are most effectively managed and preserved while benefiting livelihoods if the market-incentives of a liberalised economy are correctly in place. By examining three nationally supported payment for ecosystem service (PES) schemes in Vietnam we show how insecure land tenure, high transaction costs and high opportunity costs can undermine the long-term benefits of PES programmes for local households and, hence, potentially threaten their livelihood viability. In many cases, the income from PES programmes does not reach the poor because of political and economic constraints. Local elite capture of PES benefits through the monopolization of access to forestland and existing state forestry management are identified as key problems. We argue that as PES schemes create a market for ecosystem services, such markets must be understood not simply as bald economic exchanges between ‘rational actors’ but rather as exchanges embedded in particular socio-political and historical contexts to support the sustainable use of forest resources and local livelihoods in Vietnam.

## Introduction

Globally, environmental governance schemes are progressing with urgency to revalue and manage natural resources in support of specific social and economic goals (Li, 2007; Dressler and Roth 2011). Based on the market principles of optimal allocation and use for the common good (Goldman 2001), market-based conservation is often heralded as an efficient and effective means to improve rural livelihoods and conserve forests (Pagiola *et al*. 2005; Wunder 2005; Kumar and Muradian 2009). By assigning new market values to ecosystem services and natural resources, such schemes aim to finance their conservation over time, placing faith in the market incentives of a liberalised economy (Igoe *et al*. 2010). By creating a market for ecosystem services, strategies such as payments for ecosystem services (PES) supposedly enable local people to generate more income from harvesting fewer higher value resources than more extensive or destructive modes of production (Pagiola *et al*. 2005). Although PES was originally defined as a voluntary transaction, involving the conditional purchase of a well-defined environmental service by a buyer from a seller (Wunder 2005), most now recognise that such transactions often require states, donors and civil society to create new markets and associated institutions (Vatn 2010). Depending on how payments articulate with national, regional and local conditions, the commoditisation of ecosystem services has implications for the property rights of the targeted resources, local socio-political relations, livelihood transitions, cultural dynamics and landscape changes. Compared to other conservation interventions, the impacts of PES on livelihoods can be more pronounced over time due to the relatively focused income stream going to locals based on the imputed value of the ecosystem services they should protect. Usually held as a public good, markets commodify such ecosystem services to supposedly secure their ongoing maintenance based on agreed upon criteria that can exclude certain uses and actors (Lee and Mahanty 2009). PES-based markets thus have the potential to maintain the monetary value and function of ecosystem services by upholding regulatory objectives with productive efficiency, while excluding diverse uses of the same resource with their associated social welfare benefits (Landell-Mills and Porras 2002; Mahanty *et al*. forthcoming). In this sense, market-based approaches should be understood not simply as bald economic exchanges between rational actors with perfect market information, but rather as exchanges embedded in socio-political and cultural contexts over time and space (Landell-Mills and Porras 2002). This paper explores these processes in the context of PES implementation in Vietnam.

While Latin America has experimented extensively with diverse types of ecosystem services, markets for these services have lagged behind in Asia and Africa (Scherr *et al*. 2005). Case studies and examples from Southeast Asia are only now starting to emerge (see Clements *et al*. 2010; Soriaga and Annawi 2010; Thuy *et al*. 2010) --the first being the RUPES (Rewarding Upland Poor for Environmental Services) project (Leimona *et al.* 2008). Empirical evidence is weak on the practice and impacts of implementing PES in a centralized governance and rural livelihood context (McElwee 2012). This paper critically examines the development and implementation of three nationally supported PES schemes in Vietnam’s changing local to national political economic context. We examine the prospects for implementing PES projects under a centralised government in Vietnam, where insecure land tenure, high transaction costs, high opportunity costs and pervasive capture of benefits by local elites challenges PES policy and practice. While these factors often pre-date PES implementation, we find the convergence of PES with local political economies exacerbates unequal social relations and wealth, undermines the long-term benefits of PES programmes for rural households, and further threatens the viability of local livelihoods and conservation efforts (see McElwee 2012).

After discussing our methods, we review relevant literature on the institutional structures and livelihood impacts associated with PES schemes. Next, we describe the development of PES schemes in Vietnam at the national level, and then focus on the differential impact of PES schemes at the local level in the Ba Vi National Park. We extend this discussion to evaluate PES schemes currently being piloted in Lam Dong and Son La provinces. While piloting the schemes, the government passed a national policy on PES in the hope of strengthening forest conservation, improving local livelihoods, and generating revenue outside of the state budget for nature conservation. This paper will discuss the challenges for achieving the government’s social, economic, and environmental objectives for PES, concluding with potential implications for the scaling-up of PES schemes in the near future.

## Methods

We conducted three months of fieldwork in Ba Vi National Park in 2004–2005 and made various trips in 2009–2010 to Lam Dong and Son La provinces where the government PES policy is being piloted. Data were collected through in-depth interviews with 50 households, focus group discussions and participant observation within communities involved in the PES schemes. Project documents and government reports were analysed and project staff and policymakers were interviewed. The selection of respondents was based on their direct involvement in PES projects. Since the PES policy is still in its pilot phase in Lam Dong and Son La, a comprehensive assessment of the impacts of policy implementation would be premature. Aware of the limits of our analysis, we concentrate on local perceptions and implementation of PES schemes on the ground.

## Payment for Ecosystem Services: Theory and Practice

The idea of market-based mechanisms for conservation, and PES in particular, emerged in the 1970s from the belief that the free market, competitiveness and efficiency underpinning strong economic development could assign an appropriate market value to ecosystem services with particular functions and outcomes (Wunder 2005; Sydee and Beder 2006). In recent years, sectors of the conservation community have promoted PES as an effective market-based mechanism to ensure efficient environmental conservation amongst local users (Pagiola *et al*. 2005; Wunder 2005). Scherr *et al*. (2005) emphasize several trends that have led to the emergence of PES: (i) the increasing market valuation of ecosystem services as a means of providing local financial incentives to conservation; (ii) the search for new sources of conservation finance, particularly as a response to shortfalls in public funds; (iii) new corporate interests in environmental investment and corporate ‘social responsibility’; and (iv) supportive changes in resource governance, particularly institutional changes at the national and local level. However, as Redford and Adams (2009: 785) state, “PES is being adopted [at the national and local level] with rapid speed, and often without much critical discussion, across the spectrum of conservation policy and debate and developing a life of its own independent of its promulgators.” For these reasons, the task of critically understanding the risks and opportunities associated with PES is both urgent and significant.

PES schemes are premised on the need to price ecosystem services that are usually unvalued within markets, involving a ‘voluntary’ transaction where a well-defined environmental service (ES) (or a land-use likely to secure that service) is ‘bought’ by a ES buyer from a ES provider, if and only if the ES provider secures ES provision (conditionality) (Wunder 2005: 5). Through PES transactions, non-market values are supposedly translated into real financial incentives that pay local actors (i.e., farmers, hunter-gatherers, etc.) who are reliant on natural resources to conserve landscapes and secure ongoing provisions of ecosystem services (Engel *et al*. 2008; Corbera *et al*. 2007). The adoption of decentralized, ‘devolved’ approaches to implementing PES and related market-based mechanisms have progressed rapidly, marking a shift in resource management policies away from command-and-control to community-based, market-oriented regulation (Bird and Rodriguez 1999; Ribot 2004). Market enthusiasts argue that directly paying resource users creates incentives for them to manage and protect the natural resources and ecosystem services they already rely upon, hoping that they will manage them sustainably. PES promoters argue that the negative governance conditions associated with centralized management can be avoided if market-based mechanisms are institutionalised, devolved, and adopted by local resource users. As McCarthy and Prudham (2004) note, this has come to reflect ‘hybrid neoliberalism’ where the overlap between markets and devolved conservation centre on faith in the interdependency and flexibility of markets and civil society, such that both support the supply and demand of finances, conservation and local actions. By providing incentives in cash or in kind, PES is designed to motivate local users to adopt more sustainable land use practices that secure ecosystem services through, for example, direct management and/or improving livelihoods through alternative uses or intensification (Mayrand and Paquin 2004; Dudley *et al*. 2007). Given that PES interventions *should* generate economic returns on investment for local users, the assumption holds that the local economic benefits of such market involvement will outweigh the forgone opportunities of using natural resources for other purposes, effectively ‘paying’ for conservation (Redford and Adams 2009; Igoe *et al*. 2010).

Despite the enthusiasm for PES, some scholars now argue that market-based approaches are not a panacea for governance problems and that PES only works under certain complex environmental, socio-political and economic conditions (Landell-Mills and Porras 2002; Lee and Mahanty 2009; Muradian *et al*. 2010). In particular, PES systems require effectively functioning market mechanisms for ecosystem services, including the establishment of relationships between buyers and sellers, the creation of monitoring and regulatory mechanisms for measuring the services, and maintaining low transaction costs in pre-existing arenas of political power and economic disparity (Huang *et al*. 2009; van Hecken and Bastiaensen 2010). Crook and Clapp (1998) suggested that the success of market-based mechanisms for conservation would depend upon a good scientific understanding of ecological issues, ‘sound’ ecological knowledge by resource users, ‘strong’ management regulations and enforcement, and ‘stable’ property rights – all of which exist as a matter of degree rather than kind in developing countries. As Mahanty *et al*. (forthcoming: 3) note “PES schemes seldom resemble [an] ideal model”, with projects in areas with uncertain markets (Corbera *et al*. 2007) where buyers and sellers are in flux (Peskett *et al*. 2011), and where changing environmental services are difficult to value (Lansing 2011). While noting that many conservationists remain sceptical of PES, these authors warn an uncritical embrace of PES that neglects how politics, culture and economy govern implementation could lead to problems for local resource users and conservation efforts (Landell-Mills and Porras 2002).

The central question, then, is whether PES projects create more opportunities or risks for the rural poor. PES schemes can meet both poverty and environmental objectives only when the poor have unfettered access to forestlands providing ecosystem services that can be ‘valued’ as capital in practice. This assumption is problematic given the varied socio-cultural ties to nature and the range of socio-political and economic conditions that mediate the valuing of nature as a commodity (Dressler 2011). Scherr *et al*. (2005:535) observe that the poor are able to benefit from PES only if (a) they can provide the service at a low cost; (b) they can be contracted to protect forestlands at low transaction costs; and (c) the land use changes required for the provision of ecosystem services do not increase their labor demand or indirectly increase poverty. The potential financial benefits of PES projects to the poor are limited or non-existent if they are unable to participate in the schemes due to political constrains (e.g., corruption, etc.), or if better-off households capture most of the benefits (Lee and Mahanty 2009). Redford and Adams (2009) note that as ecosystem services become increasingly scarce and valuable, people will compete to gain control over them and the ecosystems they serve, potentially giving rise to political and economic power struggles.

Implementing PES projects potentially reinforces existing power structures, inequalities and vulnerabilities (Corbera *et al*. 2007) by changing market contexts, and exacerbating conditions that contribute to local differentiation: the ‘cumulative… process[es] of change in the ways in which different groups in rural society gain access to the products of their own or others’ labour, based on their differential control over production resources…’ (White 1989: 20). For example, as wealthier local actors work to improve the status of ecosystems through PES, they can exert their power to restrict other land use practices that poorer households invest in as vital subsistence safety-nets (see Dressler and Pulhin 2010). Increases in value of forests as a result of PES adoption may also trigger land speculation, land grabbing and land-based conflicts (Chhatre and Agrawal 2009). Across rural Southeast Asia, the trend of common holdings being partitioned and allocated as forestland contracts (Vietnam) or private title (Cambodia, Philippines) to wealthier households connected to political structures is on the rise and, as our case shows, can possibly be accelerated by PES heightening the market value of land amongst the poor with ‘insecure’ tenure, further denying them access rights to forests they depend upon for survival (Sikor and Thanh 2007; Dressler 2011; McElwee 2012). Finally, subjecting ecosystem conservation to direct market values and pricing will invariably lead to stronger commodity relations and comprehensive, landscape-scale commodification. So far, there is little evidence that these schemes are meeting the high expectations advocates place upon them (Porras *et al*. 2008), but rather a growing concern that PES could diminish rather than enhance local livelihoods (Cotula and Mayers 2009; Lee and Mahanty 2009).

## The Political Economy of PES Implementation in Vietnam: An Overview

Prior to independence, Vietnam’s forests were managed by the French colonial state. However, with the absence of the state in the uplands, in remote areas mostly local communities managed forests (Sowerwine 2004). In 1954 the post-colonial government nationalised all forests. From the 1950s until the 1980s, forests were managed by a number of State Forest Enterprises (SFE) directly under the Ministry of Forestry (currently the Ministry of Agriculture and Rural Development), and by local authorities at the district and provincial level (McElwee 2009).^1^ Often SFEs’ functions overlapped in that each was responsible for protecting forests and exploiting timber to meet logging quotas prescribed by the state. In practice, however, the SFEs focused on industrial timber exploitation disregarding their duty to protect forests (Nguyen Van Dang *et al*. 2001). As a result, local households were free to work their land in the forest for agricultural purposes, as influenced by earlier agrarian policies.^2^

By 1986, the government shifted from centralised planning to a market-oriented economy (Beresford 2008:221) – the process, known as *Doi Moi* (renovation), leading to less state control over the forestry sector and smallholder production. Subsidies previously given to state forest enterprises were soon cut off, and unproductive SFEs closed down. During the 1980s, the forest sector was in crisis. There were a number of conflicts between SFEs and local villagers over forestlands, as many villagers demanded the return of land that SFEs had monopolised. In addition, SFEs lacked investment funds to ‘renovate’ (Sikor 1998). By 1990, the country`s forest cover was 27.8%, a sharp drop from 43% in 1943 (Nguyen 2009).

In response to the crisis, the government restructured the forestry sector from forest exploitation to forest production and conservation, with an export ban to reduce the timber logging volume (McElwee 2009). In line with market reforms, the government undertook decentralization of state forestry, including passing the 1993 Land Law by which SFEs granted farmers land use certificates to lease, exchange, inherit, transfer and use land as collateral (Scott, 1999, 460).^3^ Households secured forestland title in return for signing forest protection contracts compelling them to exploit forests in a sustainable manner, particularly via replanting and protection initiatives (Sikor and Nguyen 2007). However, while partial control over the forest (and land) was now being transferred from the state to individual households, most families acquired titles to completely barren forestland (Sikor and Nguyen 2007; McElwee 2009). In some cases, SFEs emerged as powerful political actors with greater local authority than some state officials at the commune level, and resented by locals for curbing access to non-timber forest products and other subsistence resources (Sikor and Nguyen 2007: 2014). This shift started the process of smallholder ‘privatization’ and, often, more intensified production (Henin 2002).

The devolution of rights to forestlands under Doi Moi conflicted with customary tenure and associated access and use relations in the uplands. In communities with ‘shared’ access to and use of forest resources, the allocation of forestlands to individual households under title denied others their right to extract resources for subsistence, undermining traditional institutional structures and giving rise to conflicts (Sikor and Nguyen 2007). In time, the overlap of formal forestland and informal customary rights gave rise to access restrictions, unequal social and economic benefits, and livelihood marginalization (Sikor and Thanh 2007). In many instances, formal state governance structures, often associated with the SFE, still defined the monitoring and management rules of local user groups structured around forestland allocation (Sikor and Thanh 2007).

Despite significant reforms that stoked economic productivity in both rural and urban areas (Beresford 2008), land and forestry reforms were mapped onto, and partly held hostage to, the existing political economy of forest production. The political elite came to control the distribution of forestlands and the flow of surplus production from forests (Porter 1993). However, even though political hierarchies and national economic dynamics governed the distribution of benefits from forestland allocation, local peasants’ expressions of discontent (and resistance) to land allocations as infractions on their right to subsist were sufficient to allow them to actively engage and pressure state decisions as to how and to whom forestland titles were issued, and, ultimately, who became involved in payment for ecosystem service schemes (Kerkvliet 1995, 2001).

The transition to market-oriented socialism caused considerable deforestation during much of the 1990s, leading to major state-led reforestation campaigns, including Program 327 (1993–1998) and its successor Program 661 (the Five Million Hectare Program, 1999–2010), which aimed to replant more than 14.3 millions hectares by 2010 (McElwee 2009). These programs sought to protect large areas of forest in critical areas (e.g., watersheds) by issuing forestland contracts to different actors, including a large number of individual households, who were paid for protecting forests and planting trees (To 2007). Thus, the state had already drawn upon market-oriented principles to underwrite efforts to conserve forestlands and biodiversity before the adoption of current payment for ecosystem services.

The Vietnam Forestry Development Strategy 2006–2020 further reinforced the emphasis on forest protection for biodiversity conservation and environmental services in the context of existing resource uses in and around forestlands. Financial incentives were drawn from markets in order to pay users to conserve, maintain and or protect forest resources through the allocation of leases (Beresford and Fraser, 1992). The Strategy optimistically projected that by 2015 the country would derive about US $900 million from environmental services, and about US$2 billion by 2020. To facilitate the development of the market for environmental services, the government established a pilot policy framework for Payment for Forest Ecosystem Services (PFES; Decision 380) in 2008. This was the first national policy to set out basic definitions for ‘environmental service providers’ and those who benefit from forest-based ecosystem services – the ‘service users.’ The PES policy focused primarily on water supply and regulation, soil conservation, and landscape conservation for tourism purposes through local contracts. Two provinces with different forest ecosystems were selected for the pilot phase - Lam Dong in the Central Highlands and Son La in the north. Winrock International and the German Agency for International Cooperation (GIZ)^4^ were the most prominent among the international organizations that provided financial and technical support. In 2009, the total revenue derived from service buyers, mostly hydropower and water supply companies, was 77 billion Vietnam Dong (VND) (approximately US $4 million) (Hua 2010), 80% of which would supposedly be transferred to local households providing environmental services.

The results from the pilot projects in Lam Dong and Son La were used to scale up PES models to 43 forested provinces. On this basis, the government prepared a proposal for a national decree on PFES to be adopted country-wide, which was passed in April 2010. PES proponents believed that this nationwide scaling up could produce revenues of up to US $1 billion.^5^ This figure—together with State media broadcasting the positive results of the pilot projects—drove high expectations and the rapid development of PES policy and practice. In 2008, PES was recognized in the Biodiversity Law, which states that “organizations and individuals using environmental services related to biodiversity shall pay charges to service providers” (Article 74). These changes occurred in the space of only five years (Huang *et al*. 2009).

Currently, more than 20 PES projects are being implemented focusing on different kinds of ecosystem services and carbon sequestration through contracts based on existing forestland titles in the uplands. Millions of dollars in funding from both public and private sources have been committed to facilitate these initiatives for years to come. The government’s support for the rapid spread of PES is based on three social, economic and environmental objectives (Nguyen 2009): 1) PES is expected to alleviate rural poverty by helping communities to protect forests and improve local livelihoods; 2) PES will establish a sustainable source of private funding for forest protection and rural development to fill current shortfalls in the state budget; and 3) PES projects are expected to support forest protection efforts and associated ecological functions. These rapid developments in PES policies have made Vietnam a ‘successful’ model for other countries in Southeast Asia, also drawing interest from beyond the region.^6^

The first PES program we examine here is the government-supported incentive payment scheme for forest protection in Ba Vi National Park, starting in the early 1990s. The second and third are the more recent pilot schemes Lam Dong and Son La. A key difference between them is that in Ba Vi the government is the buyer and payments come from government budgets, whereas in Lam Dong and Son La the buyers are hydropower plants and water supply companies, with payments from the profits from the sale of electricity and water.^7^

## Case Study 1. Payment for Forest Protection and Planting in Ba Vi National Park

A hill station during the French colonial era, the 12,000 ha Ba Vi National Park is located in the Hanoi Metropolitan Area and Hoa Binh Province, one of seven national parks managed directly by MARD.^8^ The park borders seven communes in the buffer zone, among them Ba Vi commune. The park’s proximity to the country’s capital (80 km) has made it a popular destination for domestic tourists. After independence, the government declared the “Ba Vi Forbidden Forest” a conservation area -- an early form of national park.^9^ The area then was 2,400 ha, comprising all lands above the 400 m contour. In 1991, the government converted the Ba Vi Forbidden Forest into a National Park and enlarged it to 6,700 ha, encompassing all land above the 100 m contour. In 2003, the government further enlarged the park to approximately 12,000 ha. We focus on the period before the 2003 expansion to examine how the residents of So village in Ba Vi commune were contracted to look after the park area bordering their village.

In 1991, the Management Board of the park began contracting out forestland inside its boundaries to different groups of people, including local households, for forest protection purposes. “We were ahead of the government policy [Decree 1, issued in 1995],” said a park official in charge of forestland contracting.^10^ In effect, land was allocated for a period of 50 years, with restrictions on its use. The contracting of land in the park peaked in 1996–1997 and it was discontinued in 1999. In total, around 4,200 ha of land between 100 m and 400 m elevation were contracted to 97 land recipients. Fifty-three recipients were located in the park buffer zone, receiving 50% of the total land contracted. Decree 1 gave priority to households in the buffer zone, who were given direct monetary payments for forest protection in order to make them less reliant on forests for livelihood. Prior to 2003, annual payments for forest protection were set at 50,000 VND/ha (around US $3). Payment for forest planting was set at 1.7 million VND/ha (US $113). Since 2003, the annual payment for forest protection had increased to 100,000 VND/ha (US $5); and the payment for forest planting to 2.3 million VND/ha (US$ 153).

In Ba Vi commune, including So village, a total of 270 ha of forestland inside the park was contracted to six out of 94 households. These six households were able to access the program because of their prior knowledge of it as local officials (chairman of the Commune People’s Committee, secretary of the Communist Party and chairman of the Veteran’s Group), effectively excluding the remaining 88 households from the land contracts.^11^ The park Management Board issued land use certificates to the heads of these households, all of whom were Party officials, allowing them to keep the land for 50 years with scope for extension if they complied with forest protection regulations. Land transactions were not allowed under the Decree without permission from the Management Board and cutting trees inside the park was only permitted with special permission from the Prime Minister. In time, the 270 ha became too large for the six households to manage, so they gave annual leases of traditionally tenured swidden cultivated land to the other village households in exchange for labour for forest protection activities. Though they were aware of this practice, Park officials turned a blind eye in exchange for a share of forest protection payments from the National Park.

These events were the result of the complex nature of land tenure in Ba Vi, with different groups holding different sets of rights granted by different institutions. During colonial times, villagers’ claims to land were established through their occupation and use of land for swidden cultivation, which they were permitted to do at lower elevations, usually 100–600 m. After independence, however, the state nationalized all the forests, including those in Ba Vi, and put them under the management of State Forest Enterprises (SFEs) without ever formally recognizing villagers’ traditional claims to the land. The newly formed Ba Vi Forbidden Forest was managed by Ba Vi SFE which had a mandate to manage all the land in the area except paddy and residential land. In practice though, the SFE did not restrict local swidden cultivation,^12^ so that despite the State’s new claims on the land usurping traditional local claims, villagers continued to cultivate the land as in the past.

In 1989, the Food and Agriculture Organization (FAO) initiated a eucalyptus plantation program in Ba Vi commune aimed at increasing both local household income and forest cover. To establish the secure tenure that was a precondition for ensuring long-term benefits to households, the FAO requested the district People’s Committee to formally allocate each household 1–2 ha of land in the forests. In fact, the land formally managed by SFE and allocated to the households were former swidden plots. Households were given land title for a period of 30 years, with the possibility of extending the lease on expiry. However, with the enlargement of the forbidden forest and establishment of the national park in 1991, the villagers’ swidden land allocated under the FAO’s tree planting program now lay within the park boundary. As a result, their rights to these lands were not recognized by the national park Management Board. As the Park’s vice manager stated,

“The National Park is established according to the Prime Minister’s Decision -- the highest level of decision making power. This decision is much more powerful than the decision made by the district authority [referring to the FAO program]. So which one do you see as valid? … Villagers’ traditional claim? There is nothing to prove that. …All the land in the country belongs to the state.”^13^

Nevertheless, villagers continued to cultivate the land as before. Eventually, however, park officials prevented cropping on Park land, removing the existing crops to make way for tree planting. Villagers strongly resisted these actions by cutting down trees and letting their cattle graze freely within the forest damaging younger trees.

In sum, various actors established contrasting and overlapping claims and rights to land inside the park. This led to conflicts of various kinds occurring between the Park officials and villagers (To 2007) as the State disregarded villagers’ claims on the land, as well as unofficial and illegal deals between the six forestland contract holders and other households in the village. In addition, collusion between these six households and the park officials threatened other households’ rights to sustain existing resource use patterns. As a consequence the majority of local households failed to benefit from the FAO scheme. Local households, particularly newly established ones and recent migrants, became less likely to gain access to land, especially with formal tenure, and consequently were continually excluded from PES benefits. This situation created high transaction costs and inequities in benefit distribution from the incentive scheme.

## Case Study 2. Recent PES Schemes in Lam Dong Province

Decision 380 set an environmental service fee for hydropower plants in Lam Dong province at 20 VND (0.125 cents) per kilowatt hour of electricity produced; for water supply companies the fee was set at 40 VND (0.25 cents) per cubic meter (m3) of water supplied.^14^ The team developed a complicated equation to calculate the payment levels, but failed to find a suitable fee to apply to tourist companies benefiting from the landscape beauty of the forest. The team consulted different stakeholders, including tour operators, about their willingness to pay various fee levels and came up with VND 0.5-2% of the companies’ gross revenue.^15^ Recognizing that the forest had different qualities and ecosystem services, the team calculated payments according to the type of forest service,^16^ the status of the forest (rich, average, poor), and whether the forest was natural or planted. A K coefficient was developed to reflect these conditions.^17^ Decision 380 also stated that 10% of the total payment derived from environmental service buyers would be retained to cover the administrative costs of the government agency managing the payment, with the remaining 90% to be allocated amongst individuals, households or rural communities – the service providers – receiving forest protection contracts from the relevant state organizations.

### Implementation of Decision 380 in Lam Dong Province

Lam Dong province is located at the source of the Dong Nai river. Sixty-one per cent of the total area is covered by forest (Peter *et al*. 2009), which is of crucial importance to the level and quality of the Dong Nai river. Almost all forestlands in the province are managed by 13 state entities, such as management boards of protection and special use forests, state forest enterprises, and private enterprises renting the land for agro-forestry production and ecotourism. The remaining land is managed by 564 households, who have been allocated 2,356 ha of forest with land titles in Lam Dong (ibid.).

Decision 380 has been in force since 2008. The key activities implemented by the provincial authorities on the ground included (i) measurement of the economic value of ecosystem services, (ii) identification of buyers and sellers, and (iii) establishment of the organizational and institutional structures for the distribution of payments (Pham 2009). Around 516,800 ha of forest were identified as potentially providing ecosystem services (Peter *et al*. 2009). Water regulation, soil protection and scenic landscape preservation were determined as important services of this forest area and were economically measured according to these functions. Other services such as carbon sequestration and biodiversity conservation, which are more difficult to measure, were not accounted for during the piloting phase. A total of 13 ecosystem services buyers were identified, including two hydropower plants, two water supply agencies, and nine ecotourism companies. Payment levels were set by the government.

In the initial phase, three watershed areas were selected as pilot sites. These areas provided ecosystem services to four buyers: two hydropower plants, one water supply organization and one tourism company. The provincial Peoples’ Committee requested the buyers to pay the service fee from the beginning of 2009, generating a total annual revenue of about 47 billion VND (US $2.8 million) (Peter *et al*. 2009). Similar to the previous case, a K coefficient was used to set the different monetary values associated with the different kinds of forest services provided. Three levels of annual payments were suggested at 290,000 VND/ha (US $16) per ha for water regulation, 270,000 VND/ha (US $15) for soil protection, and 10,000 VND/per ha (US $0.5) for scenic landscape values. While the payments for water regulation and soil protection were calculated based on quality, types, and origin of forest, payments for scenic landscape beauty were based on the company’s gross revenue. To ensure that local households were able to benefit from these revenues, local authorities maintained their own contract arrangements with local households as in the 327 and 661 Programs. This meant that households without pre-existing contracts with the state forest enterprises were automatically excluded from the PES benefit stream. In many villages, the proportion of households with pre-existing contracts was as low as 10%. At the time of writing, US $2.8 million was being paid to 3,400 local households, with each contracted to look after around 20 ha of forest from the enterprises. Counting all forest environmental services provided, each household will have gained US $600/year, an amount 3.75 times higher than their income prior to PES implementation (Nguyen Truc Bong Son 2011).

Contracts in Da Nhim commune were signed between the state enterprise and individual households. However, in order to reduce transaction costs, the enterprise worked through groups of households rather than individual households. Further, the enterprise signed contracts under Decision 380 only with households who had already signed the contracts with them under Program 661, thus excluding all others from accessing the PES project. Under the PES implementation, field stations belonging to the enterprise worked with local authorities at village and commune levels to divide the forests among groups of about 9–10 households. The forest areas given to different groups varied in quality, and payments to household groups also varied. Following guidance from provincial authorities to mitigate inequality among households, in some commune villages, officials adjusted payments so that poor households in a group received higher payments per hectare than economically better off households.

The adoption of K coefficients posed a significant difficulty for local authorities. As payments were pinned to a household’s forest area and the status of its forest, changes in the latter over time made calculating payments a complex task. A general shortcoming associated with the forestland allocation process in many upland villages was that the area of land shown on the land use certificate did not match the actual area allocated (To 2007). However, lacking the budget to reconcile the data, the government instead calculated PES payments according to the land area shown in the land use certificate, and based on the forest quality at the time of land allocation.

In many areas, the implementation of PES policy is unlikely to solve the problem of poor forest governance. Payment was made based on participation of the households in the PES schemes, rather than on performance of the services provided by the forest. Furthermore, the current structure of forestry and the way in which PES schemes are implemented ensure payments do not reach the poor, particularly the poorest– usually newly-established households and migrants lacking access to land but strongly reliant on forestland and forest resources. This compromises the potential of PES schemes to provide effective forest protection and rural poverty alleviation.

## Case Study 3. Son La Pilot PES Scheme

The implementation of Decision 380 in Son La province followed a similar process as in Lam Dong. Some 397,000 ha of the total 500,000 ha of the province’s forests were brought into the payment scheme, with water regulation and soil protection being the main ecological services targeted. Three buyers were identified, including two state-owned hydropower plants and one water supply company. In contrast with Lam Dong, the forest area under the payment scheme in Son La was already allocated to more than 50,000 forest owners in the early 2000s, mainly local households. The total revenue derived from the PES in 2009 was 63 billion VND (around US $3.5 million), with an average payment per hectare of forest of 397,000 VND (US $21) (Pham 2009).

The distribution of payments in Son La faced a number of problems. Of the 63 billion VND that should have been disbursed by payment schemes in 2009, only 9 billion (14%) was transferred. Only 4,507 households, or less than 10% of households with forestland, have actually received any payment, corresponding to a forest area of 50,900 ha. A major difficulty facing authorities was the complexity of local land tenure arrangements. The distribution of payments was based on existing records of forestland allocation to households in the early 2000s. However, land allocation was implemented in a way that was “*not accurate, not complete, not scientific, too fast*” (leader of the decree drafting team, who is also the national coordinator of the pilot policy, interview, Hanoi, October 2009). Our interviews with officials from the provincial forestry department revealed similar land allocation issues to those observed in Lam Dong. The amount disbursed to individual households was based on a field-based inventory of their forest area, quality and type. However, conducting inventories is a time-consuming process, while geographical difficulties, such as no access to roads, making measurements and boundary demarcation complex and costly. The drafting decree team leader estimated that conducting an inventory of all forest areas in the province would take at least five years at a cost of 56 billion VND.

Another problem is that there have been many changes in forest area, forest status, and forest holders in the seven or eight years since forestland allocation began. Son La Province has the highest number of hydropower plants in Vietnam - currently 40, with a further 60 to be built soon. Large areas of forest in the province, most of which were already allocated to households, have been cleared for hydropower plants and many new settlements have been set up for villagers displaced as a consequence. According to interview data, changes have also resulted from the construction of two high voltage grids and a road along the provincial border. In addition, government policy has reclassified around four million ha of ‘protection forest’ to ‘production forest’ that has been converted to rubber plantations in the Central Highlands. Changes are often made without consulting local households holding land use certificates so that they actually no longer manage the land. These broader changes in land use practices and land tenure have thus complicated the processes of contracting and payment making it difficult, if not impossible, to achieve implementation in the government’s time frame.

Opportunity costs are another constraint to the success of the PES scheme in Son La. Favorable weather conditions make the province the second largest corn producing location in the country (a total of 117,000 ha, General Statistics Office 2008). Income derived from each hectare of corn averages 15 million VND per year (US $800). If these areas are preserved under a PES scheme, household income would be less than 400,000 VND (US $22). Given the high poverty rate in the province (37.9% in 2007, General Statistics Office 2008) and small area of forest that each household received (an average of two ha), most would likely opt for corn production, which as a permitted land use, generates more income than PES.

The delay with the distribution of PES payments in Son La has put pressure on the Ministry of Agriculture and Rural Development (MARD), which will most likely change the way payments are disbursed in the province. As the country’s focal point on PES, MARD reported to the Prime Minister that forest inventories for areas holding PES potential could be completed within five years. However, given the current problems faced in Son La, this time frame is highly unlikely. An alternative proposed by the Vice Minister of MARD is to instead rely on local people to accurately report their land allocations (Vice Minister Hua Duc Nhi, PFES consultation workshop, Hanoi, February 2010).

## Discussion

The Vietnamese state has been central to driving PES and the market institutions that enable the commoditization and exchange of environmental services, while also serving as a major buyer of these services. One objective driving the government was that implementing PES policy would draw significant stakeholders into forest protection, improve local livelihoods, and contribute to poverty alleviation. However, these objectives have not been fully realised by the PES pilot policy implemented in Lam Dong and Son La provinces. In addition to the state, the PES policy in Lam Dong has mobilized a significant funding pool from private actors who use the environmental services, including hydropower plants, water supply companies and tourist companies – funds which were transferred directly to local households. Where households have large landholdings (on average 20 ha), as is the case in Lam Dong, they were able to derive a substantial source of income. The incentives derived from PES schemes have motivated already locally wealthy landowners to protect the forest and secure environmental services (Peters *et al*. 2009; Nguyen Truc Bong Son 2011). However, there have been major constraints in securing long-term benefits because the forest owners in Lam Dong are state entities, such as SFEs and their management boards. The one-year protection contract signed between state entities and local households meant that household tenure over land was not secure and thus they had no guarantee of long-term benefits – an example of how overlapping tenure regimes render access and use rights ambiguous, confounding PES implementation (cf. Sikor and Thanh 2007). In Lam Dong we found that in some villages, SFEs had signed contracts only with households that retained existing contracts from the 1980s. As a result, only about 10% of village households gained access to PES benefits, whereas the remaining 90%, who held unrecognised customary tenure without a contract were excluded from benefits. This has triggered conflict in villages, with members of households excluded from benefits surreptitiously cutting coffee trees belonging to households with contracts.

Although households in Son La held more secure tenure rights than those in Lam Dong, they have also not gained any significant benefits from PES payment schemes because of their small landholdings (on average two ha). As payments were calculated on a per hectare basis, even those households with secure tenure in the province received only small sums of money from forest protection services, too small to persuade them to conserve the forest. Huang *et al*. (2009) have highlighted that for PES to work as a market-based mechanism, transaction costs must remain low and benefits must support local livelihoods. This was not the case in Son La, where a small piece of forestland was allocated to thousands of households. Furthermore, opportunity costs were high because households could derive a much higher income from corn production. This suggests that PES income may compete with, rather than supplement other livelihood activities.

Engel *et al*. (2008) observe that state structures in developing countries often have weak resource governance, high transaction costs, and information problems and that these factors could be avoided by adopting devolved market-based mechanisms. From the Vietnamese Government’s perspective, the use of PES may have served as a mechanism to reduce state budget burdens in the forestry sector, rather than as a mechanism to mobilize additional income to poor rural households. Following this logic, PES must then generate sufficient funding for forest protection, and contribute to better forest governance. However, funding is not the only barrier to effective forest governance. In Son La, weak resource governance resulted also from the complexity of land tenure, the changing status of forest resources over time, the high transaction costs in setting up agreements, and the weak capacity of the state to conduct inventories and track changes to forestland title. In Son La and Lam Dong, these same factors also impacted on the effective establishment of PES, with delays in setting up agreements and disbursing payments. The high costs of inventory and calculating acceptable payment amounts were major reasons for these delays. The government responded to these challenges with the development of a universally applicable ‘formula’ (a K coefficient) and other criteria. In Lam Dong, for instance, local authorities decided to pay better-off households a lesser amount per hectare than poor households as a levelling strategy, although both groups provided the same kinds and quality of environmental services. This suggests that while the PES schemes in Vietnam partly embrace the ideals of market-oriented solutions, the socialist market context firmly embeds these programmes within contrasting political and economic agendas. The cases presented here show that despite the shift towards the free market, the state still plays a role in mediating market activities (McElwee, 2012). In Vietnam, the state is responsible for institutional design, mediating PES contracts and, often, for buying environmental services. Despite the fact that the state-owned enterprises should run their services as financially self-sufficient, as mandated by the enterprise law, the traditional state providers – like hydropower plants and water companies using forest services – still come under strong state protection. This was reflected when the Hoa Binh hydropower plant did not pay PES fees of 218 billion VND (US$1.9 million) in 2010, despite numerous payment requests from the MARD. Eventually, MARD sent an official letter to the Prime Minister and newspapers stating the hydropower plant had ‘disregarded the Prime Minister’s order [Decision 380]. [and that] the delay in payment by the plant led to a one year delay of payment distribution to local households in Son La province’.^18^ The Hoa Binh hydropower plant was only able to do this because it is owned and protected by the Vietnam Energy Group, which belongs to the Ministry of Industry and Trade. Thus, strong state protection might lead to structural problems in the case of PES. This is particularly the case since PES schemes in Vietnam operate with the government as the sole buyer, who must respond to a range of social, political and environmental objectives.

The case studies in Lam Dong and Son La have several implications for the debate on whether PES creates opportunities or risks for the rural poor. In principle, the payments derived from PES schemes should be paid to service providers. In Vietnam, however, more than 60% of forest, particularly natural forest with many environmental services, continues to be managed by state entities.^19^ Payments derived from these services may provide a strong incentive for state entities to hold on to the land, capturing the benefit streams associated with PES while poor households are unable to access such benefits in the face of stronger restrictions on resource access. As ecosystem services become increasingly scarce and valuable, competition to gain control over the flow of services and the ecosystems that provide them will only increase – as seen elsewhere in Southeast Asia (Redford and Adams 2009; Sikor *et al*. 2010). During a workshop in Lam Dong City, December 2010, on PES in Lam Dong province, for example, a National Park director responded to a participant suggesting that some of park’s 40,000 ha of forest should be managed by local households as follows: “What is wrong with us [management board] receiving PES payment? … if we have this money we will not have to rely on state budget anymore… we will contract the land to local households if we are unable to protect the forest*.*”

The distribution of payments to households was based on formal land data records from past land allocation and forest protection programs. This meant that households established after land allocation were automatically excluded from payment schemes. This number is not small, as the land allocation process commenced in 1995 in many provinces. Clearly, when PES policy is scaled up nationally, a significant number of poor will be unable to access PES schemes and benefit from associated payments; this suggests that PES schemes operating in the existing tenure structure which privileges formal over customary rights to forest, have huge potential to marginalize poor households. Such outcomes worsen class-based disparities. In Lam Dong, for example, poorer households not benefitting from PES schemes have sabotaged the (formally tenured) coffee plantations of households qualifying for PES payments.

The PES schemes implemented in Lam Dong and Son La have similarities to earlier incentive schemes in Ba Vi National Park. In all three cases, PES payments were not reaching the poorest households and thus limiting their potential contribution to rural poverty alleviation. In Ba Vi, all payments were captured by local elites who had connections to political power through which they monopolized access to land. The scaling up of PES in areas like Ba Vi National Park will likely provide further opportunities for economically better off households, particularly those with access to political power, to capture the majority of benefits from PES schemes. The national adoption of PES will also exacerbate existing inequalities between state entities and forest-using communities because the former may continue to monopolize access to forestlands–as was done decades earlier. The simplification and de-politicization of complex resource governance structures on which PES depends may challenge the success of PES, which, in turn, can trigger tensions and conflicts centered on unequal resource access and use.

Although the Vietnamese government hoped that the introduction of PES schemes would shift the budget burden for forest protection from state to non-state actors, evidence from the three case studies indicates that this is may not occur in practice. PES-like schemes adopted in Ba Vi were entirely supported by the government. Although PES schemes in Lam Dong and Son La have been able to derive about US $60 million per year from private sector fees, there is no clear evidence that the government’s budget burden will ultimately be lowered. Instead, the implementation of PES has produced a new kind of budget burden. In Son La province, PES has come with new requirements for forest inventory procedures in the central and provincial government, which are costly and time-consuming, and for which there is limited budget and capacity. The share of payments retained by government from PES schemes was proving insufficient to cover inventory costs. New sources of funding created through PES have to be weighed against new budget burdens and the politics of allocation.,However, no new budget was created for forest protection in Ba Vi national park and in Lam Dong and Son La provinces. In addition, since most of the hydropower plants and water supply companies – the buyers of environmental services – have been government-owned, they are under the protection of the state and may delay their payments indefinitely. As a result, the government’s expectation of the national PES scheme mobilizing new private funding for forest protection has not yet been met. In any event, the central role of the state as an environmental service buyer means that the budgetary burden will be shifted within state agencies rather than reduced overall, suggesting that PES reflects as much a state-centred initiative as a market-oriented one.

The Vietnamese government also expected that the adoption of PES would improve forest protection. While PES policy in Lam Dong has decreased the number of violations, (Peters *et al*. 2009), it is still too early to conclude that a PES program would be an effective mechanism for forest protection more broadly in Vietnam. It is unclear whether forest protection in the pilot site was effective or not prior to the introduction of PES. Moreover, owing to the lack of technical capacity in measuring the quality of service provided by the forest, it is not clear if the quality of service has been improved as the result of PES implementation. In Lam Dong, most of the forest area was classified as protection forest and special use forest, and so had already been under strict state protection. In general, households have agricultural land outside the forest area managed by the state entities. As a result, pressure on forestland from cultivation practices has been much lower than in Son La and Ba Vi, where local households earn their living through cultivating forestland and it is unlikely that payments for forest protection will compete favourably with agricultural uses of that same land, at least at current payment levels.

## Conclusion

Vietnam has become known as a pioneering country in Southeast Asia for its use of market-based mechanisms to conserve natural resources. Within the country, hopes have been high that the introduction of PES as a market-based mechanism can provide a more viable and effective alternative to conventional command-and-control approaches for resource governance and sustainable development. The PES policy has been expected to broaden the benefits from forest protection in the country, improve the livelihoods of the rural poor, create sustainable financing for forest protection, and improve the ecological conditions of forests. However, the apparent PES ‘miracle’ has been hampered by a number of factors, which apply equally well to other countries in the region. Unstable land tenure introduced risks for both buyers and sellers (Wunder *et al*. 2005; Mahanty *et al*. forthcoming). The insecure nature of tenure in rural Vietnam has meant that long-term benefits from PES schemes have not been guaranteed to service providers, particularly poor households. Other countries, such as the Philippines, have struggled similarly in implementing PES, finding that the provision of secure tenure –whether private or customary, provided there is statutory recognition–is central to securing local peoples’ commitment to participating in PES schemes (Rosales 2003: 17); consequently, dramatic changes in forest status and tenure introduce further complications as market-based schemes unfold. Such factors have contributed to high transaction and opportunity costs, and ultimately challenge the likelihood of success for PES schemes.

In Vietnam, given the existing forest governance structure and the way PES payment schemes have so far been implemented, it is unlikely that the scaling up of PES programmes nationally will deliver the government’s expected outcomes. On the contrary, we argue that the implementation of PES could instead further exacerbate pre-existing inequalities as local elites monopolize direct income flows and state forest entities control the majority of national forestland. Remarkably, even after many changes in PES schemes over time, the same governance constraints and problems that have long contributed to inequities in forest access persist, with large formal forest holdings and payments going to the powerful few, while the customary land tenure of the poor remains unrecognized by state authorities so these households are unable to benefit from PES contracts. Thus far, there has been no clear evidence that PES will be an effective mechanism for the state’s priorities of socializing forest protection, improving the livelihoods of the rural poor, establishing sustainable sources of funding for the forestry sector, and improving forest quality.

## Footnotes

1 From 1955 to 1960, about 560,000 cubic meters (m3) of timber was extracted each year. From 1976 to 1980 the annual logging volume was 1.6 million m^3^ (Nguyen Van Dang *et al*. 2001)

2 While lands cleared of forest for farming centered on agricultural collectivization (Kerkvliet 1995:396), after the war in 1975, agricultural policy reforms transferred collective production to households on individual plots (Scott 1999:460).

3 The land use certificates granted 20-year renewable leases on land for annual crops and 50 year leases for perennial crops and forestland (McElwee 2009).

4 Winrock International Project, funded by USAID, works mainly to consolidate and mainstream pilot biodiversity conservation schemes, Payment for Environmental Services (PES), and livelihoods activities in Lam Dong Province. The GTZ (currently GIZ) program on management for natural resources covers three major areas: Sustainable Forest Management and Policy, Biodiversity and Climate Change and Coastal Ecosystems Program. One component of the program is to support the piloting of PES in Son La province.

5 Asia Regional Biodiversity Conservation Program, Winrock International, in a workshop in Bangkok in December 2009.

6 A senior official from the Office of the Government, a key supporter of PES development in the country, was invited to present Vietnam PES model in a major meeting on PES in Ghana in October 2009.

7 In fact, these companies are still state-owned, but in the context of renovation policy, state-owned enterprises have to be financially self-sufficient.

8 By 2006, Vietnam had 128 protected areas covering about 2.4 million ha (about 7 per cent of the total natural area of the country); 30 of them were national parks with a total area of around one million ha (Nguyen Huy Dung, 2007). Seven of these 30, which have high biological importance, are managed directly by MARD. The remaining 23 national parks are managed by Peoples’ Committee of the respective provinces.

9 The term Forbidden Forest implies the restriction in human practices such as cultivation and hunting inside the forest.

10 Interview in Ba Vi National Park, December 2004

11 “We did not know about the contracting… We only came to know about it when they [the officials who obtained the land] received money from the park.” Interview with a villager in So village in November 2004.

12 “They [the enterprise] only cared about acacia and resin tree production in the area outside the Forbidden Forest and protection of pine trees in the forbidden area” Interview with the villager in November 2004.

13 Interview, Park’s Vice Manager, Ba Vi Commune, May 2004.

14 “We had to consult much literature… but we rely very much on Russian literature on environmental economics I learned in the past [in the 1970s] for calculating the payment level… but international organizations also helped us.” Interview with team leader of the Decision 380 drafting team, Hanoi, July 2009

15 “There is no existing literature on how much the company should pay … so we suggested the figure [VND 0.5-2 per cent of the companies’ gross revenue] to the province [officials] and they said they are ok with it; we also suggested the figure to some tourist companies and they said they can afford it*.*” Interview, a team member, Hanoi, November 2009.

16 The Law on Forest Protection and Development (2004) officially classifies national forest into three types according to their ecological function. Protection forest (*rừng phòng hộ*) (accounting for 30% per cent of the total forest area), is set aside for protection of watersheds, soil and the environment. Special use forest (*rừng đặc dụng*) (10% per cent) is intended for nature conservation, protection of ecosystems and flora and fauna gene resources, and historical, environmental, and cultural sites. Production forest (*rừng sản xuất*) (60% percent) is the source of wood and forest-based products and is also meant to contribute to ecological protection.

17 The idea of a “K coefficient is to take into account variations in natural and socio-economic conditions for different types of forests in the province so that equity and fairness in duties and benefits of the services sellers can be achieved” (IGES, 2011, 19).

18 Dan Viet online newspaper is among several that covered the delay in PES fee payment by Hoa Binh hydropower plant (see Nha may thuy dien Hoa Binh chong lenh thu tuong [Hoa Binh hydropower plant disregard Prime Minister’s order] 14 April 2011, and EVN xin no 218 ti dong [Energy Vietnam asked for the delay in payment of 218 billion VND] 10 April 2011).

19 State entities include management boards of the protected areas, state forest companies, and local government at commune level. For further detail see the statistical data from the Forest Protection Department of MARD (www.kiemlam.org.vn). The monopoly of state entities over access to the forest was also hotly debated in the national PES consultation workshop organized by MARD in Hanoi on 26 February 2010.
